# Retracted: Experimental Study of Local Inner Ear Gene Therapy for Controlling Autoimmune Sensorineural Hearing Loss

**DOI:** 10.1155/2017/6809643

**Published:** 2017-07-13

**Authors:** BioMed Research International

BioMed Research International has retracted the article titled “Experimental Study of Local Inner Ear Gene Therapy for Controlling Autoimmune Sensorineural Hearing Loss” [1]. Figures 1(a) and 1(b) show identical gels other than the part of the right-hand lane that includes the band of interest in Figure 1(b).

## References

[1] Tan C.-Q., Gao X., Cai W.-J., Qian X.-Y., Lu L., Huang H. (2014). Experimental study of local inner ear gene therapy for controlling autoimmune sensorineural hearing loss. *BioMed Research International*.

